# NOD2 activation enhances macrophage Fcγ receptor function and may increase the efficacy of antibody therapy

**DOI:** 10.3389/fimmu.2024.1409333

**Published:** 2024-06-11

**Authors:** Giovanna Merchand-Reyes, Mikayla F. Bull, Ramasamy Santhanam, Maria L. Valencia-Pena, Rakesh A. Murugesan, Aadesh Chordia, Xiaokui-Molly Mo, Frank H. Robledo-Avila, Juan De Dios Ruiz-Rosado, William Edgar Carson, John C. Byrd, Jennifer A. Woyach, Susheela Tridandapani, Jonathan P. Butchar

**Affiliations:** ^1^ Division of Hematology, Department of Internal Medicine, College of Medicine, The Ohio State University, Columbus, OH, United States; ^2^ College of Medicine, The Ohio State University, Columbus, OH, United States; ^3^ Department of Biomedical Informatics, The Ohio State University, Columbus, OH, United States; ^4^ Center for Microbial Pathogenesis, Abigail Wexner Research Institute, Nationwide Children's Hospital, Columbus, OH, United States; ^5^ Kidney and Urinary Tract Center, Abigail Wexner Research Institute, Nationwide Children’s Hospital, Columbus, OH, United States; ^6^ Division of Pediatric Nephrology and Hypertension, Nationwide Children’s Hospital, Columbus, OH, United States; ^7^ Department of Surgery, The Ohio State University, Columbus, OH, United States; ^8^ Department of Internal Medicine, College of Medicine, University of Cincinnati, Cincinnati, OH, United States

**Keywords:** NOD2 agonists, monocytes, chronic lymphocytic leukemia, antibody-mediated responses, pre-clinical model

## Abstract

**Introduction:**

Therapeutic antibodies have become a major strategy to treat oncologic diseases. For chronic lymphocytic leukemia, antibodies against CD20 are used to target and elicit cytotoxic responses against malignant B cells. However, efficacy is often compromised due to a suppressive microenvironment that interferes with cellular immune responses. To overcome this suppression, agonists of pattern recognition receptors have been studied which promote direct cytotoxicity or elicit anti-tumoral immune responses. NOD2 is an intracellular pattern recognition receptor that participates in the detection of peptidoglycan, a key component of bacterial cell walls. This detection then mediates the activation of multiple signaling pathways in myeloid cells. Although several NOD2 agonists are being used worldwide, the potential benefit of these agents in the context of antibody therapy has not been explored.

**Methods:**

Primary cells from healthy-donor volunteers (PBMCs, monocytes) or CLL patients (monocytes) were treated with versus without the NOD2 agonist L18-MDP, then antibody-mediated responses were assessed. In vivo, the Eµ-TCL1 mouse model of CLL was used to test the effects of L18-MDP treatment alone and in combination with anti-CD20 antibody.

**Results:**

Treatment of peripheral blood mononuclear cells with L18-MDP led to activation of monocytes from both healthy donors and CLL patients. In addition, there was an upregulation of activating FcγR in monocytes and a subsequent increase in antibody-mediated phagocytosis. This effect required the NF-κB and p38 signaling pathways. Treatment with L18-MDP plus anti-CD20 antibody in the Eµ-TCL model of CLL led to a significant reduction of CLL load, as well as to phenotypic changes in splenic monocytes and macrophages.

**Conclusions:**

Taken together, these results suggest that NOD2 agonists help overturn the suppression of myeloid cells, and may improve the efficacy of antibody therapy for CLL.

## 1 Introduction

Antibody therapy has been successfully used to treat oncologic diseases for more than 20 years. Rituximab, the first therapeutically available antibody, is directed against CD20, expressed on B cells. Upon treatment, B cells are directly killed by antibody binding, complement and engagement of immune effector cells (1, 2). For effective elimination of the target, the activation of cells capable of effecting antibody-mediated responses is crucially needed, including both myeloid cells and natural killer (NK) cells (3, 4), which bear Fc γ receptors (FcγRs) that recognize IgG-bound targets.

The FcγR family contains multiple members that modulate antibody-mediated responses. In human monocytes and macrophages, there are three activating receptors: FcγRIa, FcγRIIa and FcγRIIIa (5, 6). Both FcγRIa and FcγRIIIa are present in the membrane in association with the common gamma chain (FcϵRIγ), which bears an immunoreceptor tyrosine-based activation motif (ITAM) (5, 7). FcγRIIa contains an intracellular tail with its own ITAM. Recognition of IgG-bound targets results in the aggregation of these activating receptors and phosphorylation of their ITAMs, initiating multiple signaling cascades, including the phosphatidylinositol 3-kinase (PI3K), mitogen-activated protein kinase (MAPKs) and NF-κB pathways (8, 9). This leads to the production of cytokines and reactive oxygen species, as well as changes in the membrane to facilitate the phagocytosis and destruction of targets. Phagocytosis itself is highly coordinated, with PI3K, RAC1, CDC42 and ARF6 localized to phagocytic cups where actin polymerization advances the cups around the opsonized targets (10).

This response is modulated by the presence of different regulators, including FcγRIIb, the FcγR with an immunoreceptor tyrosine-based inhibitory motif (ITIM) instead of an ITAM. Thus, activation of monocytes and macrophages involves a balance between ITAM and ITIM signaling (11). Modulation of this balance, for example by favoring the expression of activating receptors, is of great importance for driving stronger responses to therapeutic antibodies *in vivo* (12, 13). Due to its importance, novel therapeutic antibodies have been widely engineered to enhance the binding to FcγRs and thus activate cells in a more efficient manner (14, 15).

It is known that innate immune responses are broadly suppressed in cancer. In chronic lymphocytic leukemia (CLL), myeloid cells express higher amounts of regulatory molecules (16) and show decreased expression of some molecules related to antibody-mediated phagocytosis (17). This impedes proper activation upon antigen binding. More importantly, monocytes can differentiate into nurse-like cells (NLCs), which are macrophages widely known to support the survival and drug resistance of CLL cells (18–20). Thus, addressing the suppressive phenotype of myeloid cells is crucial to elicit better antibody-mediated responses in CLL.

To increase their anti-tumoral activity, monocytes/macrophages can be stimulated by various agonists of pattern recognition receptors (PPRs). The Toll-like receptor (TLR) 7 agonist imiquimod has been successfully used to induce myeloid cell activity in melanoma (21). The TLR8 agonist motolimod has been tested against head and neck squamous cell carcinoma in mice and humans (22, 23), and is showing preclinical efficacy against acute myeloid leukemia (AML) (24). Many other PRR agonists are currently being examined (25), while only three of them (BCG, monophosphoryl lipid A, and imiquimod) are currently used against oncologic diseases (26–28).

The potential benefits of treating CLL with PRR agonists have been previously explored. Of note, several reports support an effect of TLR9 agonists towards CLL-cell phenotypic change, increased cytokine production, and apoptosis (29–31). However, CpG 7909 in a phase I clinical trial did not result in a clear response, and testing of CpG 2006 showed that it may halt T cell proliferation (32, 33). Thus, further exploration of PRRs for CLL is needed.

The aforementioned TLRs are intracellular sensors that help detect threats inside the cell (34). Additional PRRs with this function include the nucleotide-binding oligomerization domain-containing 1 and 2 (NOD1 and NOD2) receptors, which recognize peptidoglycans found in both Gram-negative and Gram-positive bacteria (35). Muramyl dipeptide has been found to be the minimal unit that can activate NOD2, enhancing the production of humoral responses (36). Upon activation, NOD2 activates different signaling cascades including the inflammasome, NF-κB and MAPK pathways, leading to a variety of responses including cytokine production (37–39). In myeloid cells, activation of NOD2 has been linked to the production of TNFα, IL-6 and/or IL-1β (40), increased cytolytic activity against tumoral cells (41, 42), enhanced production of reactive oxygen species (43) and differentiation from classical to non-classical monocytes (44). The NOD2 agonist muramyltripeptide phosphatidylethanolamine (MTP-PE, Mifamurtide^®^) has been used therapeutically to treat osteosarcoma patients in Europe (45), while other agonists have been used for different diseases in other parts of the world (46). This supports the potential of NOD2 agonists as therapeutic adjuvants to enhance responses in myeloid cells. These agonists have also been explored for the treatment of other malignancies including AML (47, 48).

Here, we investigated the role of NOD2 activation in monocyte/macrophage antibody-mediated responses against CLL. We found that the NOD2 agonist L18-MDP activated CLL-patient monocytes, promoting the production of cytokines and an increase in the expression of FcγRs both at the transcript and protein levels. This was highly correlated to an increase in antibody-mediated phagocytosis. These effects were mediated by the NF-κB and MAPK/p38 pathways. Furthermore, we observed that NOD2 agonist treatment led to a decreased leukemic load and a shift in the phenotype of monocytes/macrophages within the Eμ-TCL1 mouse model of CLL. Finally, the agonist significantly enhanced the effects of antitumor antibody. Collectively, these results suggest that NOD2 agonists may carry potential as adjuvants for antibody therapy in CLL.

## 2 Methodology

### 2.1 Cell culture

Healthy-donor (HD) source leukocytes were purchased from Versiti Blood Services (Columbus, OH, USA). Whole-blood samples from untreated, low-count (<60,000 cells/μL) CLL patients were provided by the Leukemia Tissue Bank of The Ohio State University Comprehensive Cancer Center, obtained according to the Declaration of Helsinki and under protocols approved by the Institutional Review Board at The Ohio State University. Peripheral blood mononuclear cells (PBMCs) were isolated from each sample using lymphocyte separation medium (Corning, Corning, NY, USA), followed by density gradient centrifugation, and then washed with RPMI (Gibco, Thermo-Fisher Scientific, Whaltham, MA, USA). For PBMC experiments, cells were counted using the Cellometer Auto 2000 (Nexcelom Biosciences, Lawrence, MA, USA) and resuspended in RPMI media supplemented with 10% FBS (VWR, Radnor, PA, USA), 2 mM L-glutamine (Gibco) and 100 U/mL Penicillin-Streptomycin (Gibco) at 10x10^6^ cells/mL.

To obtain HD or CLL-patient monocytes (CD14^+^ cells), as well as CLL cells (CD19^+^), PBMCs were incubated with magnetic CD14^+^ or CD19^+^ selection beads (Miltenyi Biotec, Waltham, MA, USA) for 15 minutes on ice. Following this, cells were centrifuged and resuspended in MACS buffer (Miltenyi Biotec) and added to an LS selection column. After three washes, cells were recovered from the column, washed with incomplete RPMI, counted, and resuspended at 3x10^6^ cells/mL in supplemented RPMI media.

For stimulation, cells were plated and treated with L18-MDP (MDP) (InvivoGen, San Diego, CA, USA). A concentration of 1 µg/mL was selected after testing a concentration-response curve in isolated monocytes, and is similar to a previous report (49). Cells were incubated at 37°C, 5% CO_2_, for 24 hours unless indicated otherwise. For inhibition experiments, monocytes were treated with DMSO (as inhibitor vehicle control) or: Bay 11-7085 at 5 µM (Millipore Sigma, St. Louis, MO, USA), trametinib at 10 nM (Selleck Chemicals, Houston, TX, USA), or SB202190 at 1 µM (Selleck Chemicals) for 30 minutes before MDP stimulation. Dimethyl sulfoxide (DMSO; Sigma Aldrich, St. Louis, MO, USA) was used to prepare stock solutions and the final concentration of DMSO was under 1 μL/mL.

### 2.2 Quantitative real-time PCR

Cultured cells were collected and then mRNA extracted using the Norgen Total RNA extraction kit (Norgen Biotek, ON, Canada), according to the manufacturer’s instructions. Collected mRNA was quantified using the nanodrop ND100 (Thermo-Fisher Scientific), and then reverse transcribed using the high-capacity reverse transcriptase kit (Applied Biosystems, Thermo-Fisher Scientific) into cDNA. Then, quantitative real-time PCR (qPCR) was done using SYBR green (Applied Biosystems) and the QuantStudio™ 3 machine (Life Technlologies). RCN (relative copy number) values were calculated for each target, normalizing to a GAPDH housekeeping control according to the equation RCN = 2 ^(-ΔCt)^ x 100, where ΔCt = Ct_problem_ – Ct_GAPDH_) (50). Primers used are shown in **Table 1**.

**Table 1:** Quantitative real-time PCR primers used.

	Forward	Reverse
GAPDH	ACTTTGGTATCGT GGAAGGACT	GTAGAGGCAGGGATG ATGTTCT
FcγRIa	GGCAAGTGGACAC CACAAAGGCA	GCTGGGGGTCGAGG TCTGAGT
FcγRIIa	TTGCTGCTGCTGG CTTCTGC	GTAGCTGGGCTGCG TGTGGG
FcγRIIb	TGACTGCTGTGCT CTGGGCG	AGCCTTTGGGGGA GCAGGTGT
FcγRIIIa	CCTCTCCACCCTCA GTGACCCG	TGGAGCAACAGCCA GCCGAT
FcϵR1γ	CAAGCAGCGGCC CTGGGAG	TTCCTGGTGCTCA GGCCCGT

### 2.3 Immunoblot analysis

Cells were collected and lysed using 70 µL TN1 lysis buffer for every 1 x 10^6^ cells, as previously described (51). Protein was quantified using the *DC* Protein Assay according to manufacturer’s instructions (Bio-Rad, Hercules, CA, USA). Samples were boiled with 5x SDS sample buffer (150 mM Tris; 11.5% SDS; 0.05% bromophenol blue; 50% glycerol; 1% 2-mercaptoethanol). Samples were then size-separated using SDS-PAGE. Proteins were transferred to a nitrocellulose membrane using the Trans-Blot Turbo Transfer System (Bio-Rad). Membranes were blocked with the LI-COR blocking solution (LI-COR Biotechnology, NE, USA) and incubated with primary antibodies overnight at 4°C in LI-COR antibody diluent. The following primary antibodies were utilized: phospho-p42/44 (polyclonal, product #9101; or clone D13.14.4E; Cell Signaling Technology), phospho-MAPKAPK2 (polyclonal, product #3041; Cell Signaling Technology), phospho-p38 (clone D3F9; Cell Signaling Technology), phospho-p65 (clone 93H1; Cell Signaling Technology) mouse anti-human GAPDH (clone D4C6R; Cell Signaling Technology), rabbit anti-human FcϵRIγ (product 06-727; Upstate, Sigma-Aldrich), and mouse anti-human calreticulin (clone FMC 75, Enzo Life Sciences, Farmingdale, NY, USA). After probing with the corresponding fluorescently-conjugated secondary antibody (IRDye 800CW goat anti-rabbit, or IRDye 680RD goat anti-mouse antibodies; LI-COR) for 1 hour at room temperature, membranes were developed using the Odyssey CLx (LI-COR). For densitometry analysis, Fiji was used (52).

### 2.4 Flow cytometry

After PBMC or monocyte stimulation, cells were collected and washed with PBS. Then, cells were transferred into v-bottom plates and incubated with whole human IgG (Jackson ImmunoResearch Labs, West Grove, PA, USA) at 10 µg/mL in PBS for 15 minutes on ice. After Fc block, the specific antibody cocktail was added (**Table 2**). Samples were acquired using the LSR-Fortessa (BD Biosciences) at the Flow Cytometry Shared Resource at The Ohio State University Comprehensive Cancer Center and analyzed using FlowJo version 10.7.2 (Ashland, OR, USA). **Supplementary Figure 1** shows an example of the gating used in monocyte analysis.

**Table 2:** Flow cytometry panels.

Panel	Cocktail
Panel 1: Monocyte activation	CD14 Alexa Flour 647 (clone HCD14, BioLegend) CD38 FITC (clone HIT2, BioLegend CD163 PeCy7 (clone GHI/61, BioLegend) CD86 PE (clone BC96, BioLegend) HLA-DR PercP (clone L243, BioLegend) Live/Dead Blue (Thermo-Fisher Scientific)
Panel 2: T cell activation	CD3 Pacific Blue (clone OKT3, BioLegend) CD4 Brilliant Violet 711 (clone RPA-T4, BioLegend) CD8 PercP (clone SK1, BioLegend) CD69 Alexa Fluor 488 (clone FN50, BioLegend) CD25 PeCy7 (clone BC96, BioLegend) CD86 APC (clone IT2.2, BioLegend) Live/Dead Blue
Panel 3: B and NK cell activation	CD19 PeCy7 (clone HIB19, Invitrogen) CD69 Alexa Flour 488 CD86 APC CD56 Super Bright 600 (clone NCAM, Invitrogen) CD107a PE/Dazzle (clone H4A3, Invitrogen) Live/Dead Blue
Panel 4: FcγRs	CD64 (FcγRIa) PercP Cy5.5 (clone 10.1, BioLegend) CD32a (FcγRIIa) FITC (clone IV.3, StemCell Technologies, Vancouver, Canada) CD32b (FcγRIIb) PeCy7 (clone S18005H, BioLegend) CD16 (FcγRIIIa) Brilliant Violet 711 (clone 3G8, BioLegend) Live/Dead Blue
Panel 5: CLL cells (Mouse model)	CD45 Brilliant Violet 421 (clone 30-F11, BioLegend) CD19 PE (clone 1D3/CD19, BioLegend) CD5 Alexa Fluor 647 (clone 53-7.3, BioLegend) Live/Dead Blue.
Panel 6: Monocyte/macrophage phenotype (Mouse model)	CD45 Brilliant Violet 421 CD11b Brilliant Violet 711 (clone M1/70, BioLegend) Ly6G PerCP (clone 1A8, BioLegend) Ly6C PeCy7 (clone HK1.4, BioLegend) F4/80 Brilliant Violet 605 (clone BM8, BioLegend) IA-IE Brilliant Violet 785 (clone M5/114.15.2, BioLegend) iNOS PE (clone CXNFT, Invitrogen) EGR2 APC (clone Erongr2, Invitrogen) Live/Dead Blue

### 2.5 ELISA

Cultured cells were centrifuged, and cleared supernatants collected and used for ELISA with the Human TNFα DuoSet ELISA kit (R&D Systems, Minneapolis, MN, USA) according to manufacturer’s instructions. For acquisition, the BioTek Synergy HTX plate reader was used (Agilent, Santa Clara, CA, USA).

### 2.6 Antibody-mediated binding (rosetting)

To determine antibody-mediated binding of targets, opsonized sheep red blood cells (sRBCs; Colorado Serum Company, Denver, CO, USA) were prepared as previously described (53). sRBCs where washed with PBS until the supernatant appeared clear. Then, 2 μL of PKH26 Red Fluorescent Dye (Sigma-Aldrich), diluted in Diluent C, were added to the cell pellet and thoroughly mixed. The reaction was stopped with a 1:1 volume of FBS, and sRBCs were washed with PBS until clear. Fluorescent sRBCs were then opsonized with rabbit anti-sheep antibody (Sigma-Aldrich) on ice for 2 hours before washing.

Upon monocyte stimulation with MDP at 1 μg/mL for 24 hours, cells were used for rosetting analysis, as previously described (53). Briefly, monocytes were divided and incubated with 1 uL of packed, opsonized or non-opsonized, fluorescent sRBCs. Cells were mixed and spun down, then incubated on ice for 1 hour. After incubation, cells were fixed with 1% paraformaldehyde in PBS for 15 minutes before analysis. The number of bound targets per 100 monocytes, as well as the percent of active cells (monocytes with bound sRBCs) were counted through microscopy using the Olympus BX41 (Olympus Lide-Science/Evident, Tokyo, Japan). Samples were counted in a blinded fashion by two different subjects. The number of bound non-opsonized targets in control samples was subtracted from the number of bound opsonized targets.

### 2.7 Antibody-mediated phagocytosis

sRBCs were stained with the pH sensitive Protonex Red 600 dye (AAT Bioquest, Pleasanton, CA) at 4 μM in Hanks Buffered Saline Solution (HBSS; Gibco). The staining was done for 30 minutes at 37°C and then sRBCs were washed 3 times with PBS. sRBCs were then opsonized with antibody as stated above.

For measuring phagocytic activity, monocytes were treated with DMSO or pathway inhibitors for 30 minutes before stimulation with MDP at 1 μg/mL for 24 hours. Then, cells were washed and stained for contrast with carboxyfluorescein succinimidyl ester (CFSE, Thermo-Fisher Scientific) at 0.5 μM in PBS for 10 minutes at room temperature; the reaction was stopped with FBS and cells spun down. Cells were resuspended in complete media to perform the assay. Monocytes were mixed with 1 μL of packed, fluorescently-labeled, opsonized sRBCs for 45 minutes at 37°C, then washed and fixed with paraformaldehyde at a final concentration of 1%. The amount of phagocytosed sRBCs was counted per 100 cells under the microscope, and the number of cells with ingested events was also recorded as active phagocytes. The counts were done independently by two or three different subjects in a blinded fashion and averages calculated. As control, phagocytosis of non-opsonized, fluorescent sRBCs was checked for negativity. Some donors and results are shared among different phagocytosis assays.

### 2.8 Mouse model

Mice were housed in a vivarium at The Ohio State University, following all institutional guidelines. All experimental procedures were approved by The Ohio State University Institutional Animal Care and Use Committee.. An adoptive transfer Eμ-TCL1 mouse model of CLL was used (54, 55). Briefly, 1x10^7^ splenocytes from CLL-burdened Eμ-TCL1 mice were used to engraft C57BL/6 mice. After 2 weeks of disease development, treatments (5 mg/kg of MDP and/or 1 mg/kg αCD20 (Invivogen, San Diego, CA, USA, catalog #mcd20-mab10 -) were delivered intraperitoneally three times weekly, for 2 weeks. Engrafted C57BL/6 mice, left untreated, and nongrafted C57BL/6 mice served as controls. Mice were euthanized using a CO_2_ chamber, followed by cervical dislocation. Spleens and peripheral blood were collected. For spleens, the tissue was weighed and disrupted using a 100 μm cell strainer (Corning); erythrocytes from both blood and disaggregated spleens were eliminated by centrifugation using lymphocyte separation medium. Cells were washed, resuspended in PBS and used for flow cytometry staining.

For flow cytometry staining, cells were placed in a v-bottom plate and stained as above using the antibodies listed for Panels 5 and 6 (**Table 2**). For monocyte and macrophage phenotyping (Panel 6, iNOS and EGR2), we used the Foxp3 Intracellular staining kit (Thermo-Fisher Scientific) according to the manufacturer’s instructions, as described previously (56–58). For acquisition, Absolute Count Beads (BioLegend) were used.

### 2.9 Statistical analysis

Graphs were prepared using Excel (Microsoft Corporation, Redmond, WA). For two-group comparisons, a one-tailed t-test analysis for paired samples was performed using Excel. For multi-group comparisons, data were analyzed with the mixed effects model using SAS 9.4 (SAS Institute, Cary, NC, USA). Statistical analyses included only the engrafted groups (vehicle, MDP, αCD20 and combination) for mouse experiments, nongrafted controls were not compared. Data in graphs is shown as mean + standard deviation (S.D.).

## 3 Results

### 3.1 NOD2 agonists influence the phenotype of monocytes and enhance FcγR-mediated phagocytosis

**Figure 1:**
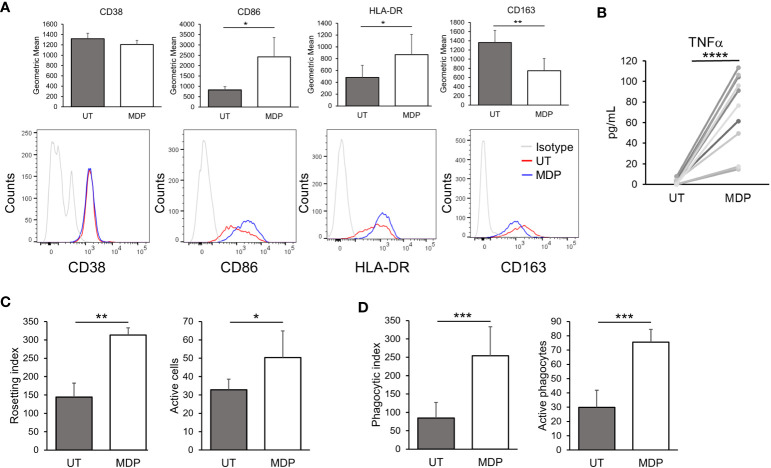
NOD2 agonists enhance monocyte FcγR-mediated phagocytosis. **(A)** HD PBMCs were treated with MDP at 1 μg/mL for 24 hours. Cells were collected, and the indicated markers were evaluated in CD14^+^ cells by flow cytometry (n=3). Top graphs show the expression levels by geometric mean of fluorescence intensity (GMFI); lower panels show representative histograms. **(B)** Isolated monocytes were treated with MDP at 1 μg/mL for 24 hours; supernatants were obtained and production of TNFα was quantified by ELISA (n=10). **(C, D)** Monocytes were treated with MDP at 1 μg/mL for 24 hours. Then, antibody-mediated functions were analyzed: **(C)** Rosetting (n=3) and **(D)** Phagocytosis (n=7). In both cases, the index indicates the number of sRBCs bound/engulfed by 100 monocytes, while the number of active monocytes indicates the percent of active cells (having surface bound or engulfed sRBCs). *p ≤ 0.05, **p ≤ 0.01, ***p ≤ 0.001, ****p ≤ 0.0001.

Monocytes and macrophages are well-known responders to NOD2 agonists (40–42). To confirm this, we first treated PBMCs from HDs with MDP at 1 μg/mL for 24 hours. Flow cytometry was then used to measure different activation markers in monocytes and lymphocytes to determine which populations show an efficient response to NOD2 agonists. As shown in **Figure 1A**, monocytes showed a significant increase in CD86 and HLA-DR. Concurrently, there was a significant decrease in the M2-associated marker CD163. These results suggest that monocyte activation was skewed toward a proinflammatory phenotype. In T cells, we found that NOD2 agonist treatment increased CD86 in CD4^+^ T cells, which may suggest a slight activation of this population, as has been suggested previously (59) (**Supplementary Figure 2A**). B and NK cells were also examined, but only a reduction in NK-cell CD107a was observed (**Supplementary Figures 2C, D**, respectively). Since changes on monocytes were significant, we decided to further test the effects of NOD2 in isolated monocytes. 24 hours following stimulation of NOD2, monocytes produced robust levels of TNFα, measured in the supernatants (**Figure 1B**). These results confirm earlier reports that monocytes are activated by NOD2 agonists (40, 49).

We next tested whether NOD2 stimulation would increase the phagocytic ability of monocytes, specifically in the context of FcγR-mediated responses. For this, we treated monocytes with MDP at 1 µg/mL for 24 hours and then incubated them with fluoresceinated, antibody-opsonized sRBCs. Here, cells were subjected to rosetting (initial binding stage of phagocytosis) and ingesting assays. Rosetting was done by incubating monocytes with the sRBCs at 4°C while incubation at 37°C was done in parallel to measure ingestion. As shown in **Figure 1C**, there was a significant increase in antibody-mediated binding (rosetting) in agonist-treated monocytes compared to controls, which is also reflected in the number of cells with bound sRBCs. Likewise, the number of ingested, opsonized sRBCs was significantly higher in treated monocytes (**Figure 1D**). There was also a significant increase in the number of actively phagocytosing monocytes. Taken together these results indicate that NOD2 stimulation leads to an increase in FcγR-mediated phagocytosis.

### 3.2 NOD2 stimulation modulates FcγR expression

**Figure 2:**
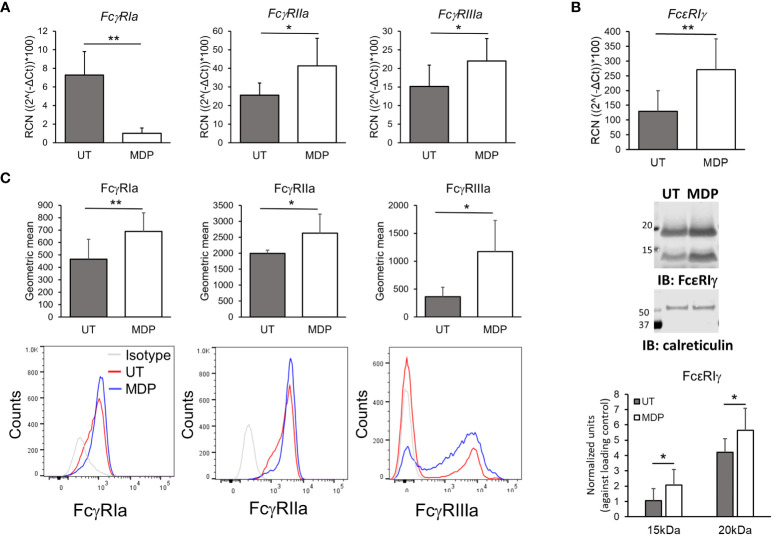
NOD2 stimulation modulates FcgR expression. Monocytes were treated for 24 hours with MDP at 1 mg/mL. Cells were collected to analyze **(A)** transcripts (n=5) and **(B)** surface expression (n=6) of the FcgRs by qPCRor flow cytometry, respectively. Bottom histograms show a representative donor. **(C)** mRNA and total protein level of the common gamma chain (FcεRIγ) were evaluated through qPCR and western blot (n=5). *p ≤ 0.05, **p ≤ 0.01.

To test whether the increase in phagocytosis could be accounted for by a change in FcγR expression, we isolated monocytes from HDs and treated for 24 hours with vehicle or MDP at 1 µg/mL. Following this, RNA was isolated and transcripts for FcγRIa, FcγRIIa, FcγRIIIa, FcϵRIγ and FcγRIIb (the inhibitory FcγR) were evaluated by qPCR. Results (**Figure 2A**) showed that there was a significant increase in the expression of FcγRIIa and FcγRIIIa. Contrary to our expectation, however, RNA expression of FcγRI was significantly lower in MDP-treated monocytes. Transcripts of FcϵRIγ were significantly increased by MDP treatment (**Figure 2B**, upper panel). Changes in FcγRIIb expression were not significant (**Supplementary Figure 3A**).

We also measured surface expression of FcγRs by flow cytometry. Consistent with an increase in transcripts, there was a significant increase in surface expression of FcγRIIa and FcγRIIIa in isolated monocytes after 24 hours with NOD2 agonist treatment (**Figure 2C**). Surprisingly, the surface expression of FcγRI was also significantly increased despite the observed reduction in transcript (**Figure 2C**). In addition to the increase in the geometric mean of expression of the activating FcγRs, we also observed a slight but significant increase in the percent of monocytes positive for FcγRI, as well as an increase of the monocyte population expressing FcγRIIIa (**Supplementary Figure 4**). This indicates a phenotypic change into intermediate or non-classical monocytes after NOD2 stimulation (60).

Because surface expression of FcγRI depends on FcϵRIγ (7, 61), we also measured protein levels of FcϵRIγ using immunoblot analysis under the same stimulation conditions. Of note, we detected two bands when measuring FcϵR1γ, which has been seen by other authors previously and may correspond to either dimers or phosphorylation of the protein (7, 62); thus, densitometry analysis was done for both. As shown in **Figure 2B** (middle panel and lower-panel graph), MDP significantly increased protein levels of FcϵRIγ. Hence, despite reducing the transcript of FcγRI, MDP treatment led to greater surface expression of all three activating FcγRs on monocytes.

### 3.3 NF-κB and p38 are required for NOD2-mediated effects on FcγR

**Figure 3:**
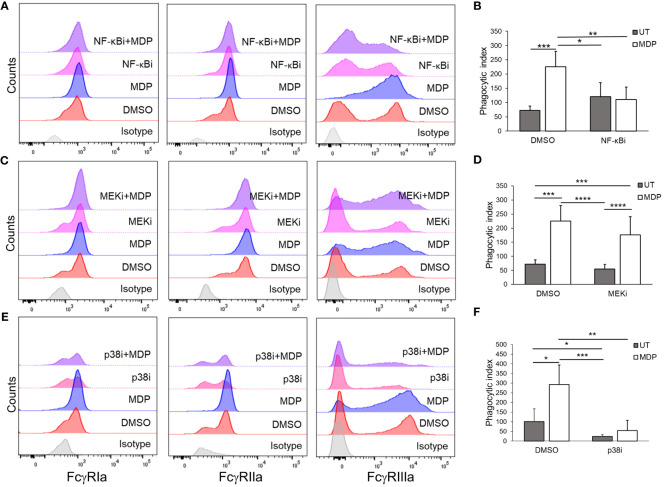
NF-kB and p38 are required for NOD2-mediated effects on FcγR. Monocytes were treated with inhibitors against **(A, B)** NF-kB, **(C, D)** MEK, **(E, F)** or p38 for 30 minutes, before stimulation with MDP for 24 hours. Following incubation, **(A, C, E)** expression of the FcγRs was measured through flow cytometry (representative histogram of n ≥ 4 is shown); **(B, D, F)** antibody-mediated phagocytosis was evaluated (n ≥ 3). *p ≤ 0.05, **p ≤ 0.01, ***p ≤ 0.001, ****p ≤ 0.0001.

Previous studies have demonstrated that NF-κB, MEK and p38 are activated downstream of NOD2 (37–39). We therefore explored which of these pathways was required for MDP-mediated changes in FcγR expression and function. For this, HD monocytes were isolated and each of these three pathways was inhibited using pharmacologic inhibitors prior to treating the monocytes with MDP. To verify efficacy of the inhibitors, immunoblot analysis was performed with phospho-specific antibodies for NF-κB, ERK and MAPKAPK2 (downstream of p38), measured at 24 hours post-treatment (**Supplementary Figure 5**). [Of note, no visible activation of p38 was detected at this time point after MDP stimulation, which may be due to p38 activity peaking earlier after stimulation (63–65)]. qPCR and flow cytometry were done to measure FcγR expression. As shown in **Figure 3A** and quantified in **Supplementary Figure 6**, MDP-driven increases in FcγR surface expression were prevented by inhibition of NF-κB and p38 but not by inhibition of MEK. Changes in transcript after MDP were also largely prevented by inhibition of NF-κB and p38 (**Supplementary Figure 7**). Consistent with the effects of these inhibitors on changes in FcγR expression, the MDP-mediated increase in phagocytosis was prevented with NF-κB and p38 inhibition (**Figure 3B**). Taken together, these data demonstrate that NF-κB and p38 are required mediators for enhancing FcγR expression and function downstream of NOD2.

### 3.4 NOD2 regulates FcγR function in CLL-patient monocytes

**Figure 4:**
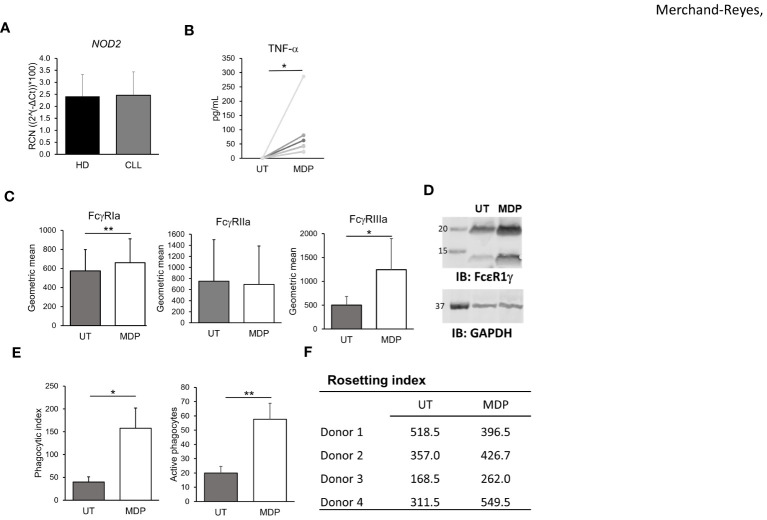
NOD2 regulates FcγR expression function in CLL-patient monocytes. **(A)** Levels of NOD2 expression in HD and CLL-patient monocytes were measured by qPCR (n=5 donors each). CLL-patient monocytes were stimulated for 24 hours with MDP at 1 μg/mL and **(B)** production of TNFα was evaluated in the supernatant (n=8); **(C)** levels of FcγRs were measured by flow cytometry (n=6) or **(D)** FcϵRIγ abundance was observed by western blot (representative blot, n=3 donors). **(E)** Antibody-mediated phagocytosis (n=3), or **(F)** Rosetting (n=4) were also measured. *p ≤ 0.05, **p ≤ 0.01.

CLL-patient monocytes are known to have altered phenotype compared to HDs (17). Thus, to test whether the effects of NOD2 stimulation seen in HD monocytes were reproducible in CLL-patient monocytes, we isolated monocytes from deidentified peripheral blood samples obtained from CLL patients and measured NOD2 expression using qPCR. As shown in **Figures 4A, B**, NOD2 was expressed in CLL-patient monocytes at similar transcript levels to HD monocytes. As with HD monocytes, CLL-patient monocytes were able to respond to MDP by producing TNFα after 24 hours of stimulation. Further, MDP treatment of CLL-patient monocytes also enhanced surface expression of FcγRI and FcγRIIIa, observed by flow cytometry (**Figure 4C**), and expression of FcϵRIγ, measured by immunoblot (**Figure 4D**). In addition, the transcriptional levels of FcγRIIb in CLL-patient monocytes remained unchanged after NOD2 stimulation (**Supplementary Figure 3B**). However, in contrast to HD monocytes, MDP treatment did not increase surface expression of FcγRIIa in CLL-patient monocytes after MDP treatment (**Figure 4C**, middle graph). However, consistent with an overall increase in activating FcγRs, MDP treatment of CLL-patient monocytes led to a significantly higher level of phagocytosis (**Figure 4E**). In contrast to HD monocytes, the same increase in rosetting was not seen in patient monocytes (**Figure 4F**), although monocytes from 3 of the 4 donors showed higher levels with MDP treatment. This suggests that despite a lower overall response than that seen in HD monocytes, CLL-patient monocytes are capable of responding to MDP by increasing FcγR expression, cytokine production and phagocytic ability.

In addition to monocytes, the effect of MDP on CLL cell activation and proliferation in patient samples was examined, as this could be a potential and undesirable side effect. PBMCs from CLL patients were treated with MDP for 24 hours, then expression of CD86 in CLL cells was measured using flow cytometry. Although there was some increase in CD86, it was not significant (**Supplementary Figure 8A**). We next isolated B/CLL cells and treated with vehicle or MDP, counting cell numbers and viability at different time points. As shown in **Supplementary Figures 8B, C**, the viability of CLL cells was not affected by MDP. These results suggest that NOD2 activation does not affect CLL cell viability. Taking cell number and percent viability together, results also suggest that MDP did not directly affect CLL-cell proliferation.

### 3.5 NOD2 activation enhances effects of antitumor antibody *in vivo*

**Figure 5:**
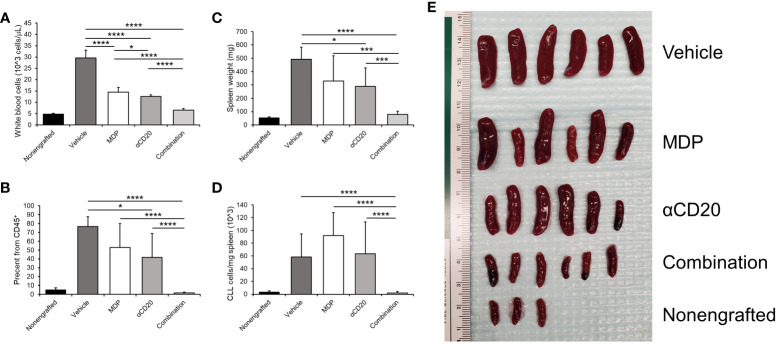
NOD2 activation enhances effects of antitumor antibody *in vivo*. Mice were engrafted with splenocytes from a diseased Eμ-TCL1 mouse and disease was allowed to develop for two weeks. Mice were treated with MDP, αCD20 antibody or a combination three times per week for two weeks. Mice were then euthanized, and blood and spleen samples obtained. **(A)** White blood cell counts were taken as explained in the methods. **(B)** Percent CD19^+^CD5^+^ cells in CD45^+^ peripheral blood was measured by flow cytometry and graphed. **(C)** Spleen weights were taken, and **(D)** number of CD45^+^CD19^+^CD5^+^ cells per milligram spleen weight was measured (n=3 for nonengrafted, n=6 for all others). (**E**) Picture depicting the spleens from the indicated groups is shown. *p ≤ 0.05, ***p ≤ 0.001, ****p ≤ 0.0001.

To test the effects of NOD2 stimulation on FcγR function *in vivo*, we used a mouse model that mimics human CLL (66, 67). C57/BL6 mice were engrafted with splenocytes from diseased Eμ-TCL1 mice, as described in the methodology. Disease was allowed to develop for 2 weeks. Animals were either treated with vehicle, treated with MDP, treated with a suboptimal dose of αCD20 antibody or treated with MDP plus αCD20 antibody in combination. Mice were sacrificed after 2 weeks of treatment. Healthy, nonengrafted mice were included as controls. CLL loads in peripheral blood and in the spleens were assessed. Results showed that white blood cell (WBC) counts in peripheral blood were significantly reduced by each single treatment, and dual treatment led to greater reduction than either single treatment (**Figure 5A**). There was also a significantly lower percentage of CD5^+^/CD19^+^ CLL cells after antibody treatment, and dual treatment led to a lower percentage than either single treatment (**Figure 5B**).

In addition to the leukemic load in the blood, we analyzed the spleen, as CLL cells tend to accumulate in this organ (68). Spleen weights were also significantly lower in mice treated with antibody, but MDP plus antibody led to spleen weights significantly lower than those for untreated or for either single treatment (**Figure 5C**). The number of CLL cells per milligram of spleen weight (**Figure 5D**) was also significantly lower with dual treatment compared to either single treatment or vehicle control. Furthermore, this was also observed in the percent of CD19^+^CD5^+^ cells from the viable CD45^+^ population (data not shown). An image of the spleens is shown in **Figure 5E**. These results suggest that the combination of NOD2 agonist plus therapeutic antibody may be more effective than antibody alone.

### 3.6 NOD2 agonists shift monocyte/macrophage phenotype *in vivo*

In addition to the leukemic burden, we tested whether MDP combined with antitumor antibody could modulate the phenotypes of monocytes and macrophages *in vivo.* For this, cells from the spleen were obtained and stained for flow cytometry to detect M1- and M2-related markers on monocytes and macrophages. Cells were gated as CD45^+^CD11b^+^Ly6G^-^ and further classified by expression of MHC-II and/or F4/80 as previously done (56). Percentages of cells expressing iNOS (M1) or EGR2 (M2) (58) were measured to assess M1 or M2 polarization. Results from nonengrafted, healthy control mice were also collected.

**Figure 6:**
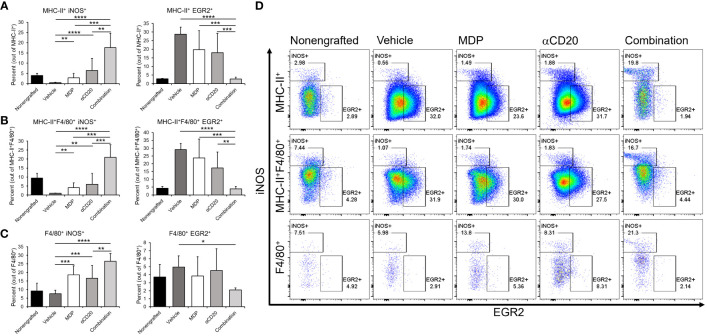
NOD2 agonists shift monocyte/macrophage phenotype in vivo. C57BL/6 mice were engrafted with splenocytes from a diseased Em-TCL1 mouse and the disease was allowed to develop for two weeks. Mice were treated with MDP, aCD20 antibody or a combination three times a week for two weeks. Then, mice were sacrificed, and spleen samples obtained. Cells were stained for flow cytometry and monocytes/macrophages were identified as CD45+CD11b+Ly6Gcells and divided by F4/80 and/or MHC-II expression. **(A)** MHC-II+, **(B)** MHC-II+F4/80+ and **(C)** F4/80+ cells were then characterized as M1 by iNOS expression, or as M2 by EGR2 expression. Percents of M1 or M2 in the different populations are shown. **(D)** Representative dot plots from one mouse per group for iNOS/EGR2 expression are shown (n=3 for unengrafted, n=6 for all others). **p ≤ 0.01, ***p ≤ 0.001, ****p ≤ 0.0001.

In MHC-II^+^ populations either negative or positive for F4/80 (mainly monocytes or macrophages, **Figures 6A, B**, respectively) there was a significant upregulation of iNOS with either single treatment, and dual-treatment led to significantly higher M1 marker iNOS than either single treatment or vehicle. Neither single treatment significantly downregulated the percent of myeloid cells expressing the M2 marker EGR2, but dual-treatment significantly reduced EGR2 compared to vehicle control and compared to either single treatment. In the F4/80^+^ group (**Figure 6C**), all treatments led to significantly higher iNOS versus vehicle, and combination treatment led to significantly higher iNOS than antibody-alone treatment.. Taken together, these data suggest that MDP/antibody combination treatment not only decreases CLL burden but also shifts myeloid phenotypes towards a more proinflammatory, and potentially M1-like state.

## 4 Discussion

In the present study, we report for the first time the potential for NOD2 agonists to offset the suppressive leukemic microenvironment and enhance antibody-mediated responses in CLL. MDP led to enhanced responses *in vitro* in monocytes from CLL patients, although these responses do not entirely mimic the observations seen in HD cells. This may be related to the overall altered phenotype of myeloid cells seen in CLL, and the role they have as leukemia-supportive cells (16–20, 56). Of note, MDP significantly enhanced the activity of αCD20 antibody *in vivo*, leading to lower tumor burden and a shift towards a proinflammatory phenotype in monocytes and macrophages.

The activation of monocytes in both HD and CLL-patient samples after NOD2 stimulation led to a significant increase in the expression of activating FcγRs. Previously, it has been shown that NOD2 promotes a phenotypic change from classical to non-classical monocytes, which involves an increase of FcγRIIIa (44). This observation is in line with ours and, in addition, we also saw a significant increase in the surface expression of FcγRIa, likely being stabilized by the common gamma chain (61). In combination with our observations of increased expression of HLA-DR and CD86, along with lower expression of CD163, these results suggest a shift towards an intermediate phenotype of monocytes after NOD2 stimulation (60). The increase in expression of activating FcγRs in the membrane is of importance, since patients with active CLL have a dominance of FcγRIIb signaling, which can lead to decreased antibody-mediated activity (5, 69, 70). In addition, FcγRs are crucial for αCD20 antibody efficacy *in vivo* (69, 71). Thus, using a NOD2 agonist in combination with a therapeutic antibody may overcome resistance to the antibodies. This is supported further by the increase in phagocytosis seen in both HD and CLL-patient monocytes, which is one of the main mechanisms for elimination of αCD20-opsonized targets by myeloid cells, and has been observed *in vivo* in B cell lymphoma (72). Further experiments are ongoing to elucidate the effects of NOD2 stimulation in NLCs from CLL patients.

We also observed that NOD2 stimulation may not only influence antibody-mediated responses, but also myeloid cells within the microenvironment. Using the adoptive transfer Eμ-TCL1 mouse model, we saw a significant decrease of the disease burden in mice treated with the combination, in comparison with either single treatment, both in circulation and in the spleen. Most importantly, we also observed an increase of the percent of monocytes or macrophages that expressed iNOS. This effect was even greater when mice were treated with both MDP and αCD20 antibody, compared with either treatment alone. In addition, the combination treatment was the only one that reduced the percent of EGR2^+^ monocytes and macrophages, suggesting that dual treatment was needed to overcome the suppressive environment. Of note, NOS2 or iNOS have been widely attributed to M1-related IFNγ stimulation, while higher EGR2 expression is highly correlated with M2-like responses (57, 58). Thus, the iNOS response to dual treatment suggests an active switch in the phenotype of myeloid cells.

There are different mechanisms that may play a role in the change in monocytes’/macrophages’ phenotypic differences, as well as the reduced leukemic burden, which are observed in the combination treatment. First, the combination may result in an increase in FcgRs as seen in human patient samples, or perhaps other factors involved in downstream signaling cascades are shared between the two processes. For example, the downstream mediator RIP2 has been shown to be common between NOD2 and FcγR, although knockout of RIP2 did not lead to defects in IgG2a-driven FcγR signaling (73), and the mouse αCD20 antibody used in the current study was based on IgG2a. In addition, signals like NF-κB or MAPK happen through activation of FcγRs or NOD2, while an increase in IFNγ production has been suggested for NOD2 mediated stimulation (74). Further research is currently in progress to delineate the mechanism of action of the combination.

It has been previously suggested that NOD2 activation may result in a variety of outcomes in myeloid cells, depending on the agonists used (42). Mifamurtide® has been shown to induce monocyte activation in treated patients, (reviewed in (45)), which involved production of cytokines and direct cytotoxicity against tumoral cell lines (75–77). In addition, macrophages polarized *in vitro* with GM-CSF and further activated with MTP-PE and IFNγ are capable of inhibiting osteosarcoma growth by producing soluble factors, although independent of TNFα or IL-1β (78). Dieter et al. showed that MTP-PE could induce TNFα and nitric oxide from liver macrophages and that MTP-PE-stimulated macrophages showed direct cytotoxic effects against the P815 mouse mastocytoma cell line (79). Our observation of TNFα production following MDP treatment further supports these observations, while translating our observations to other diseases and potentially other diseases may be carefully considered.

There is concern that MDP may have effects apart from promoting myeloid-cell activation. A previous report showed that MDP increased activation and survival of CLL cells in a specific group of patients, which may be an undesired side effect (80). However, we did not see a substantial activation of B cells in HD or CLL patients (as measured by CD86 expression), either as isolated cells or in PBMC cultures. In line with these observations, MDP alone does not increase the leukemic load *in vivo*. Although results may differ due to the use of different stimulation approaches, our observation suggests that the use of NOD2 agonists for CLL treatment does not result in leukemic progression.

Along with this, the benefits of NOD2 agonists seem to outweigh such potential unwanted effects when used against other types of cancer. Mifamurtide® has been used for the treatment of osteosarcoma in Europe. In 2009, the European Medicines Agency granted Takeda marketing approval, based on the INT-0133 clinical trial (45). The trial examined the effects of MTP-PE with chemotherapeutic agents, finding that MTP-PE plus a four-drug chemotherapy regimen (doxorubicin, ifosfamide, cisplatin and methotrexate) showed the best event-free survival (81). Further studies have been done to complement observations from the INT-0133 trial (82). The use of this NOD2 agonist is approved for youth populations (2 to 30 years of age) as follow-up treatment after surgical removal of the main affected tissue (45).

In conclusion, the present study provides evidence that combination therapy of αCD20 antibody and NOD2 agonists may be useful to overcome antibody resistance and to promote the elimination of leukemic cells in CLL. This may also be of benefit for patients who acquired resistance to BTK inhibitors or to other therapies. Further research is required to elucidate the specific mechanisms by which NOD2 agonists reverse suppression in myeloid cells, especially in combination with FcγR activation.

## Data availability statement

The raw data supporting the conclusions of this article will be provided openly by the authors upon request.

## Ethics statement

The studies involving humans were approved by The Ohio State University Institutional Review Board under OSU-1997C194. The studies were conducted in accordance with the local legislation and institutional requirements. The participants provided their written informed consent to participate in this study. The animal study was approved by The Ohio State University Institutional Animal Care and Use Committee. The study was conducted in accordance with the local legislation and institutional requirements.

## Author contributions

GM-R: Formal analysis, Investigation, Project administration, Visualization, Writing – original draft, Writing – review & editing. MFB: Investigation, Visualization, Writing – original draft, Writing – review & editing. RS: Investigation, Visualization, Writing – review & editing. MV-P: Investigation, Visualization, Writing – review & editing. RM: Investigation, Visualization, Writing – review & editing. AC: Investigation, Visualization, Writing – review & editing. X-MM: Formal Analysis, Visualization, Writing – review & editing. FR-A: Investigation, Resources, Writing – review & editing. JR-R: Investigation, Resources, Visualization, Writing – review & editing. WC: Conceptualization, Resources, Writing – review & editing. JCB: Conceptualization, Resources, Writing – review & editing. JW: Methodology, Resources, Writing – review & editing. ST: Conceptualization, Funding acquisition, Supervision, Writing – original draft, Writing – review & editing. JPB: Conceptualization, Funding acquisition, Supervision, Writing – original draft, Writing – review & editing.

## Supplementary material

Supplementary Figure 1 General gating strategy for HD or CLL-patient monocytes. For evaluating the expression of the FcγRs in monocytes by flow cytometry, viable cells were selected by **(A)** first cleaning up doublets (singlet selection). **(B)** Then, gating was done to exclude debris in the SSC vs FSC dot plot. **(C)** All cells were stained for viability, so nonviable cells were excluded following SSC vs FSC gating. For monocytes, the geometric mean fluorescence or selection of FcγR-positive populations were done with this sub-gate, subtracting the isotype control signal. A similar strategy was used for evaluation of activation-related proteins in PBMCs, where selection of monocytes, T, B and NK cells was done after **(C)**, staining for population-specific proteins. Depicted is an example healthy-donor monocyte sample.

Supplementary Figure 2 Effects of NOD2 stimulation in other immune-cell populations. Isolated PBMCs were treated with MDP at 1 µg/mL for 24 hours. Then, samples were collected, and the indicated markers were analyzed by flow cytometry in viable **(A)** CD3^+^CD4^+^, **(B)** CD3^+^CD8^+^, **(C)** CD19^+^, and **(D)** CD56^+^ cells (n=3). *p < 0.05, **p ≤ 0.01.

Supplementary Figure 3 MDP treatment does not significantly affect FcγRIIb expression. Monocytes from HD (**A**; n=5) or CLL patients (**B**; n=7) were treated with MDP at 1 μg/mL for 24 hours. Then, levels of FcγRIIb expression were measured by qPCR. Results are shown as relative copy numbers (RCN).

Supplementary Figure 4 NOD2 agonists increase the expression of activating FcγRs. HD monocytes were treated with MDP at 1 μg/mL for 24 hours. Then, cells were collected and evaluated for surface expression of FcγRs. Percentages of positive cells for **(A)** FcγRIa, **(B)** FcγRIIa, and **(C)** FcγRIIIa are shown (n= 6). Top graphs show averages +S.D., while histograms in the bottom panels show a representative donor. *p ≤ 0.05, **p ≤ 0.01.

Supplementary Figure 5 Verification of inhibitor activity. Monocytes were treated with inhibitors against **(A)** NF-κB, **(B)** MEK or **(C)** p38. To ensure appropriate blocking, the activation of downstream targets was assessed by western blot. Of note, cells were collected to measure levels of protein phosphorylation 24 hours after stimulation with MDP. Figure shows a representative blot for each inhibitor (n ≤ 3). GAPDH was used as loading control.

Supplementary Figure 6 Inhibition of NF-κB and p38 affects monocyte FcγR transcriptional responses to NOD2. Healthy-donor monocytes were treated with inhibitors for **(A)** NF-κB, **(B)** MEK and **(C)** p38 before stimulation with NOD2 agonist for 24 hours. Cells collected, and total RNA obtained. The expression of FcγR was evaluated through qPCR. *p ≤ 0.05, **p ≤ 0.01, ***p ≤ 0.001, ****p ≤ 0.0001 (n ≤ 3).

Supplementary Figure 7 Inhibition of NF-κB and p38 affects NOD2-mediated changes in FcγR surface expression in monocytes. HD monocytes were treated with inhibitors for **(A)** NF-κB, **(B)** MEK and **(C)** p38 before stimulation with NOD2 agonist for 24 hours. Cells were collected and the expression of FcγR was evaluated through flow cytometry. Expression was calculated by the geometric mean; the percent of FcγRIIIa positive population is also shown. *p ≤ 0.05, **p ≤ 0.01, ***p ≤ 0.001, ****p ≤ 0.0001 (n ≥ 3).

Supplementary Figure 8 MDP stimulation does not induce CLL cell activation and survival *in vitro*. **(A)** PBMCs from CLL patients were isolated and stimulated with 1 μg/mL MDP for 24 hours. Then, cells were collected and the expression of CD86 in B cells was evaluated by flow cytometry (n=3). Isolated B cells from CLL patients were treated once with MDP at 1 μg/mL; then, the number of **(B)** viable cells and the **(C)** percent viability was evaluated at the indicated time points (n=3).
