# Supplementary figures and images for: NOD2 activation enhances macrophage Fcγ receptor function and may increase the efficacy of antibody therapy
Source: Front Immunol. 2024 Jun 11;15:1409333. doi: 10.3389/fimmu.2024.1409333 (PMC11196781; doi:10.3389/fimmu.2024.1409333)

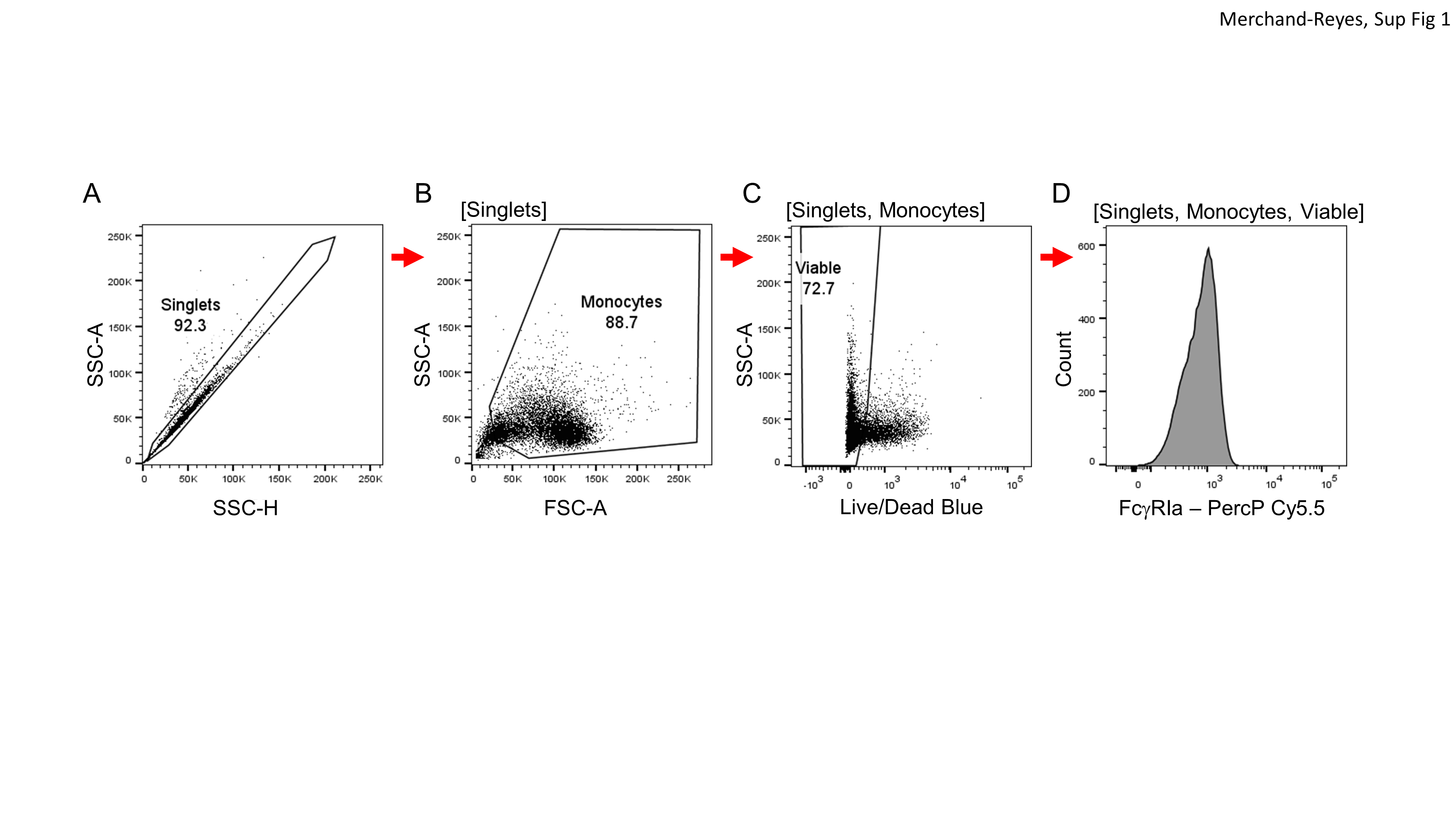

Supplement: Supplementary Figure 1 — General gating strategy for HD or CLL-patient monocytes. For evaluating the expression of the FcγRs in monocytes by flow cytometry, viable cells were selected by (A) first cleaning up doublets (singlet selection). (B) Then, gating was done to exclude debris in the SSC vs FSC dot plot. (C) All cells were stained for viability, so nonviable cells were excluded following SSC vs FSC gating. For monocytes, the geometric mean fluorescence or selection of FcγR-positive populations were done with this sub-gate, subtracting the isotype control signal. A similar strategy was used for evaluation of activation-related proteins in PBMCs, where selection of monocytes, T, B and NK cells was done after (C), staining for population-specific proteins. Depicted is an example healthy-donor monocyte sample. [file Image_1.tif]

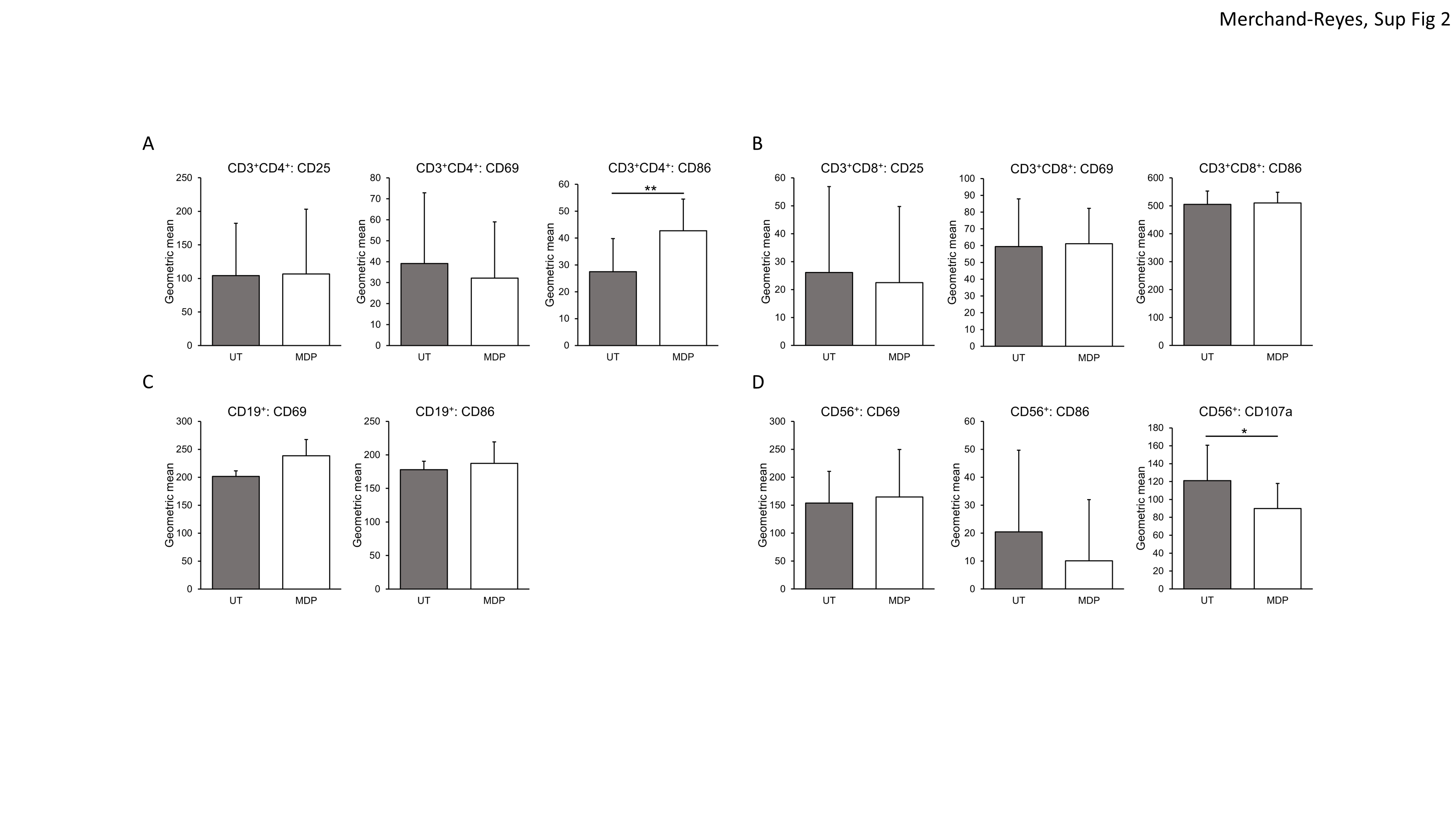

Supplement: Supplementary Figure 2 — Effects of NOD2 stimulation in other immune-cell populations. Isolated PBMCs were treated with MDP at 1 µg/mL for 24 hours. Then, samples were collected, and the indicated markers were analyzed by flow cytometry in viable (A) CD3+CD4+, (B) CD3+CD8+, (C) CD19+, and (D) CD56+ cells (n=3). *p < 0.05, **p ≤ 0.01. [file Image_2.tif]

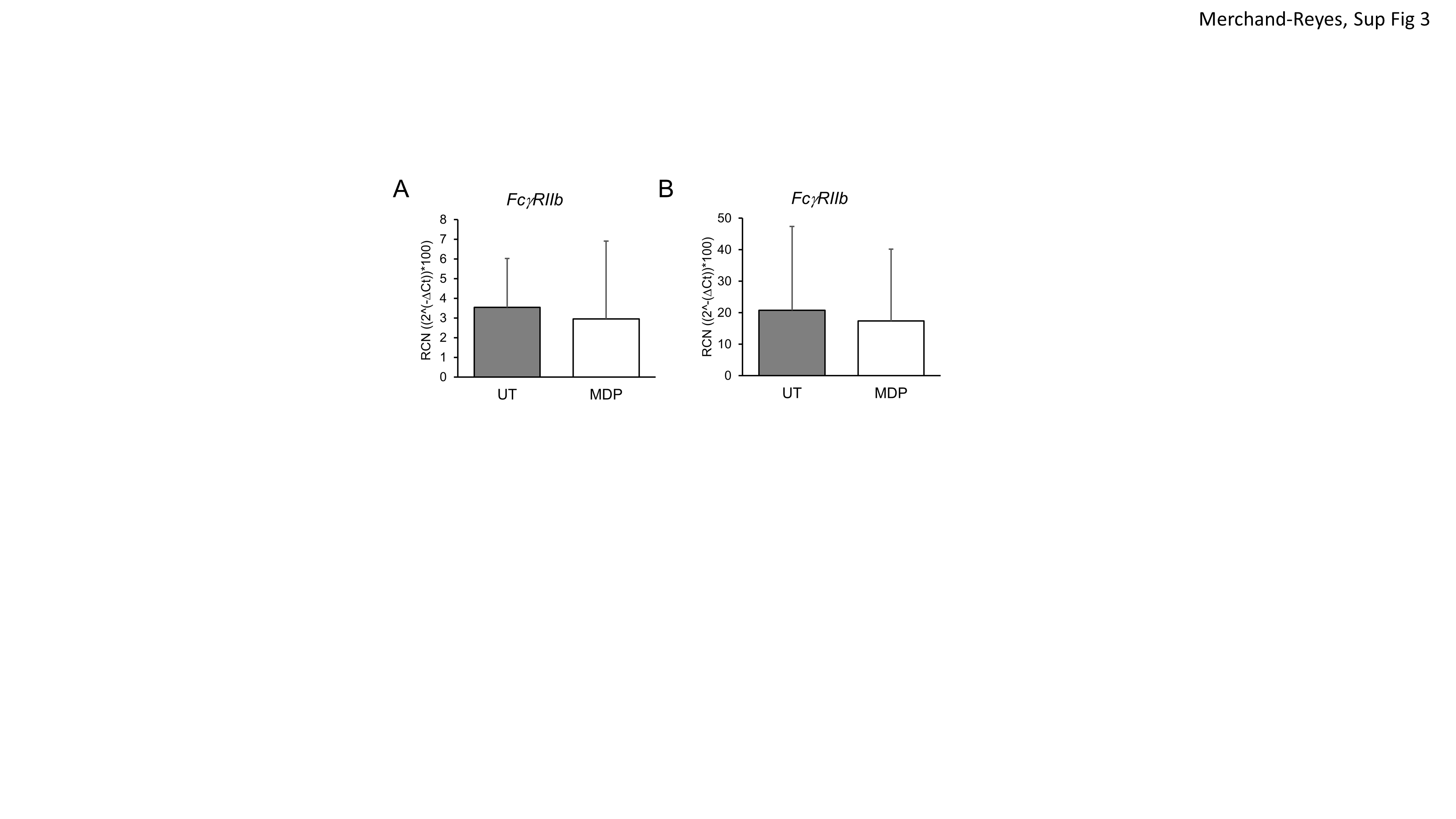

Supplement: Supplementary Figure 3 — MDP treatment does not significantly affect FcγRIIb expression. Monocytes from HD (A; n=5) or CLL patients (B; n=7) were treated with MDP at 1 μg/mL for 24 hours. Then, levels of FcγRIIb expression were measured by qPCR. Results are shown as relative copy numbers (RCN). [file Image_3.tif]

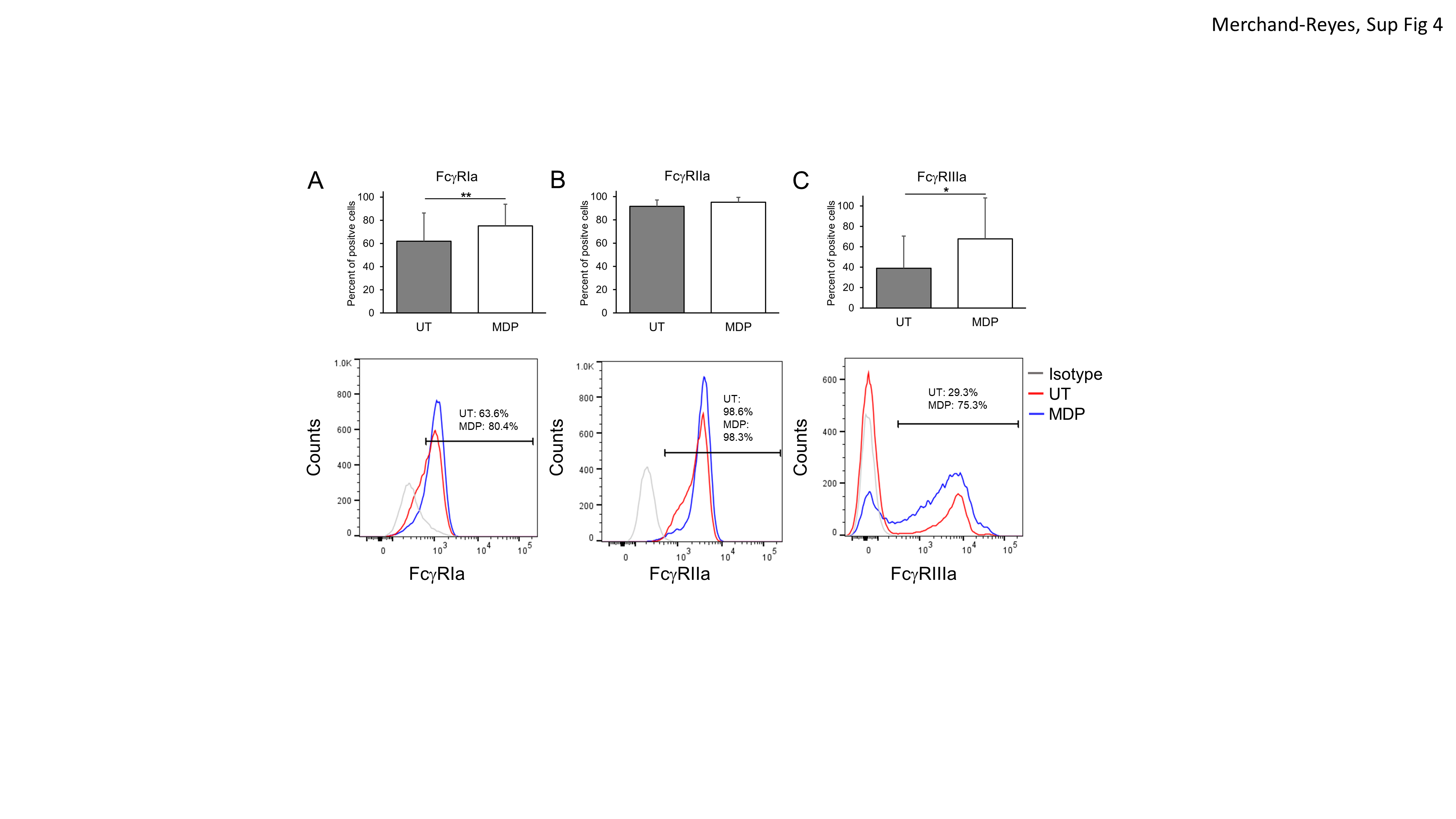

Supplement: Supplementary Figure 4 — NOD2 agonists increase the expression of activating FcγRs. HD monocytes were treated with MDP at 1 μg/mL for 24 hours. Then, cells were collected and evaluated for surface expression of FcγRs. Percentages of positive cells for (A) FcγRIa, (B) FcγRIIa, and (C) FcγRIIIa are shown (n= 6). Top graphs show averages +S.D., while histograms in the bottom panels show a representative donor. *p ≤ 0.05, **p ≤ 0.01. [file Image_4.tif]

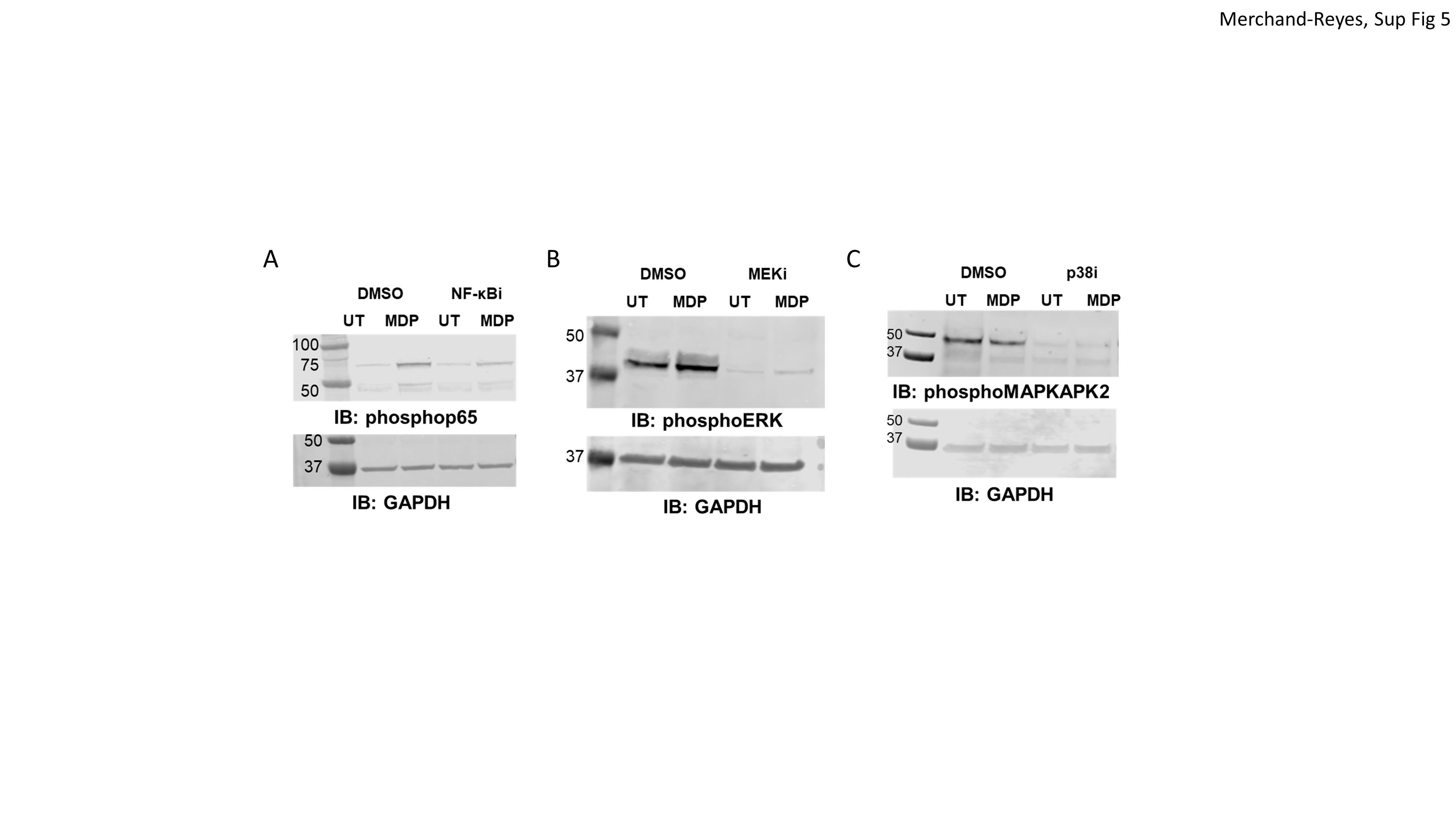

Supplement: Supplementary Figure 5 — Verification of inhibitor activity. Monocytes were treated with inhibitors against (A) NF-κB, (B) MEK or (C) p38. To ensure appropriate blocking, the activation of downstream targets was assessed by western blot. Of note, cells were collected to measure levels of protein phosphorylation 24 hours after stimulation with MDP. Figure shows a representative blot for each inhibitor (n ≤ 3). GAPDH was used as loading control. [file Image_5.tif]

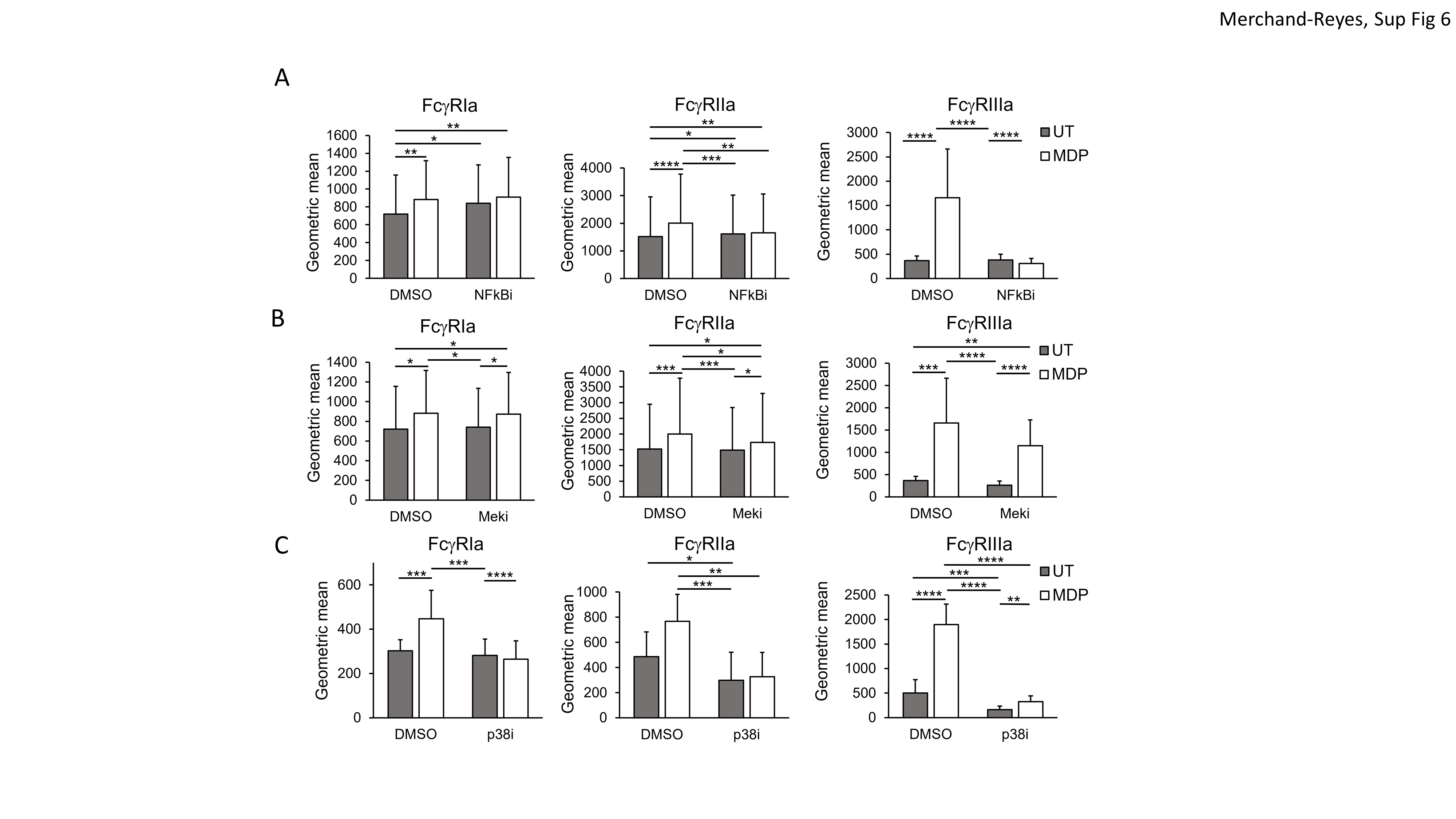

Supplement: Supplementary Figure 6 — Inhibition of NF-κB and p38 affects monocyte FcγR transcriptional responses to NOD2. Healthy-donor monocytes were treated with inhibitors for (A) NF-κB, (B) MEK and (C) p38 before stimulation with NOD2 agonist for 24 hours. Cells collected, and total RNA obtained. The expression of FcγR was evaluated through qPCR. *p ≤ 0.05, **p ≤ 0.01, ***p ≤ 0.001, ****p ≤ 0.0001 (n ≤ 3). [file Image_6.tif]

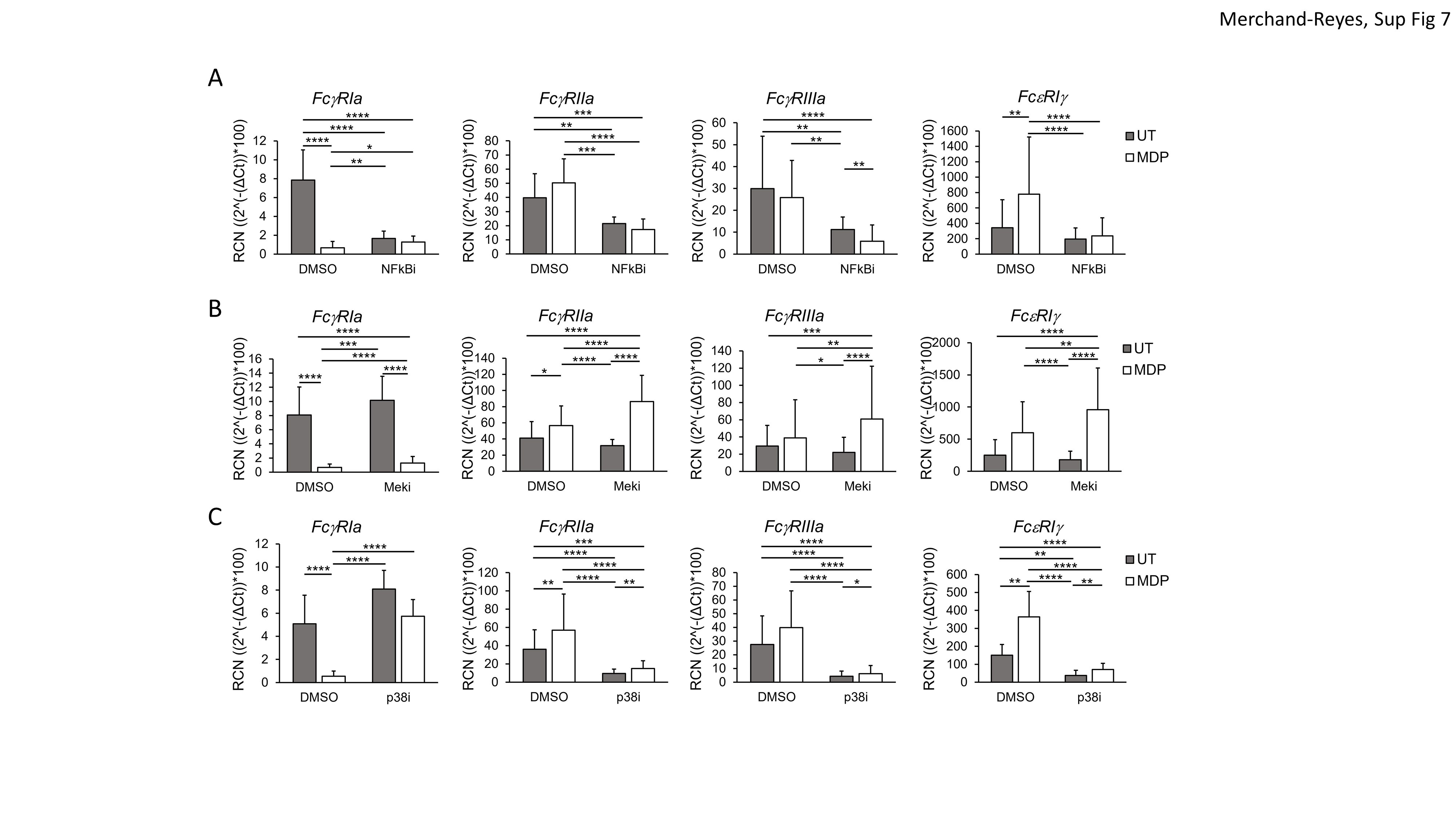

Supplement: Supplementary Figure 7 — Inhibition of NF-κB and p38 affects NOD2-mediated changes in FcγR surface expression in monocytes. HD monocytes were treated with inhibitors for (A) NF-κB, (B) MEK and (C) p38 before stimulation with NOD2 agonist for 24 hours. Cells were collected and the expression of FcγR was evaluated through flow cytometry. Expression was calculated by the geometric mean; the percent of FcγRIIIa positive population is also shown. *p ≤ 0.05, **p ≤ 0.01, ***p ≤ 0.001, ****p ≤ 0.0001 (n ≥ 3). [file Image_7.tif]

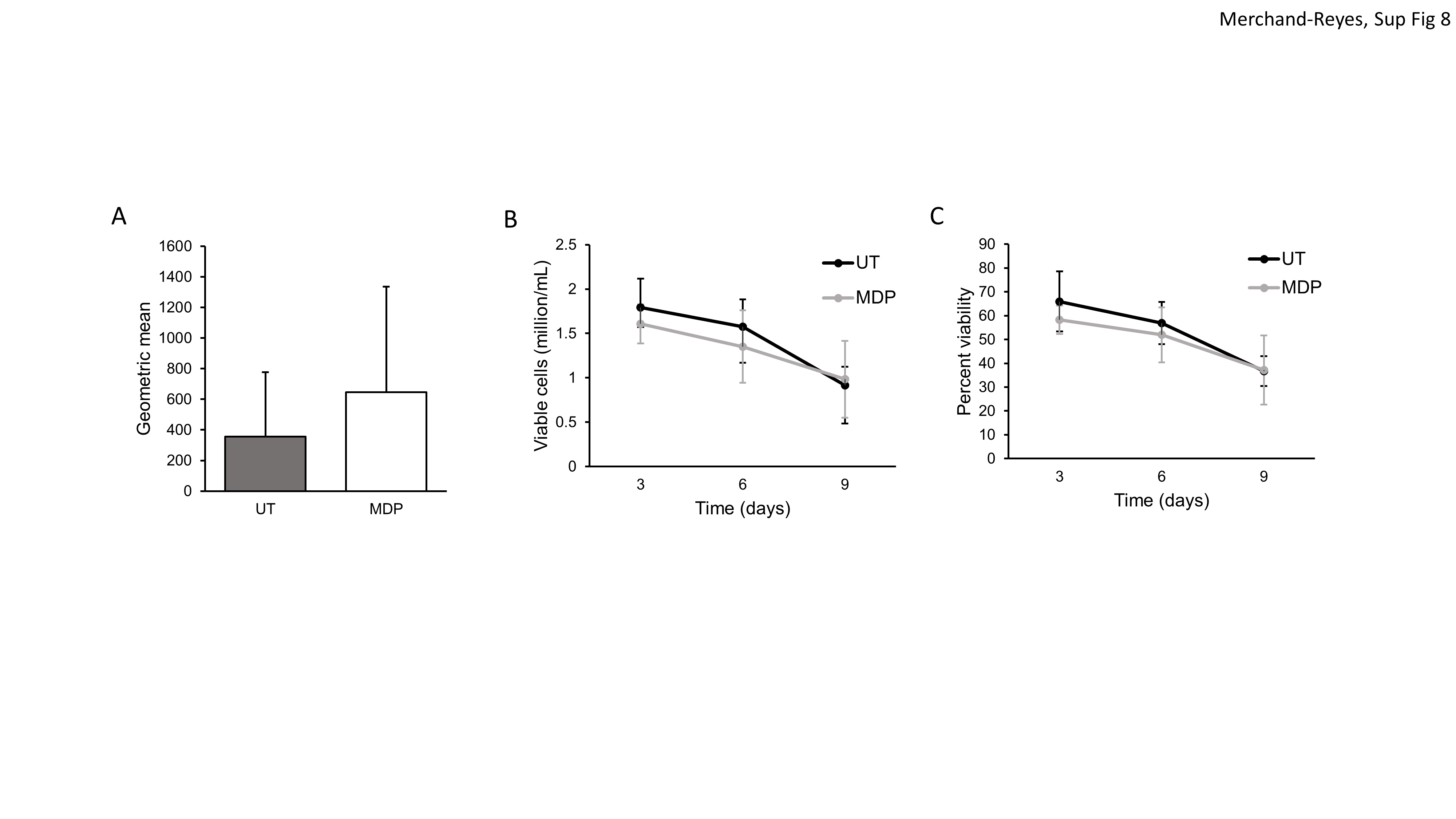

Supplement: Supplementary Figure 8 — MDP stimulation does not induce CLL cell activation and survival in vitro. (A) PBMCs from CLL patients were isolated and stimulated with 1 μg/mL MDP for 24 hours. Then, cells were collected and the expression of CD86 in B cells was evaluated by flow cytometry (n=3). Isolated B cells from CLL patients were treated once with MDP at 1 μg/mL; then, the number of (B) viable cells and the (C) percent viability was evaluated at the indicated time points (n=3). [file Image_8.tif]
